# 

**Published:** 1910-08

**Authors:** 


					﻿Vol. XXVI.
AUGUST 1910.
No. 2.
TEXA
MEDICAL
■■JOURNAL
“Red Back”
PUBLISHED AT AUSTIN, TEXAS, BY
F. E. DANIEL, M. D„ - - -
PRINTED BY THE VON BOECKMANN«>N	]
Subscription, $1.00 a Year, in Advance. -	-	-	- Single Copies, 10 Cents
Entered at the Postoffice at Austin, Texas, as second class mail matter.
				

